# Effects of Mumps Outbreak in Hospital, Chicago, Illinois, USA, 2006

**DOI:** 10.3201/eid1603.090198

**Published:** 2010-03

**Authors:** Amanda L. Bonebrake, Christina Silkaitis, Gaurav Monga, Amy Galat, Jay Anderson, JoEllyn Tiesi Trad, Kenneth Hedley, Nanette Burgess, Teresa R. Zembower

**Affiliations:** University of Illinois at Chicago, Chicago, Illinois, USA (A.L. Bonebrake); University of Chicago Hospitals, Chicago (A.L. Bonebrake); Northwestern Memorial Hospital, Chicago (C. Silkaitis, G. Monga, A. Galat, J. Anderson, J.T. Trad, K. Hedley, N. Burgess, T.R. Zembower)

**Keywords:** Mumps, MMR, outbreak, epidemiology, electronic medical records, hospital, Chicago, USA, research

## Abstract

In 2006, nearly 6,000 mumps cases were reported in the United States, 795 of which occurred in Illinois. In Chicago, 1 healthcare institution experienced ongoing transmission for 4 weeks. This study examines the outbreak epidemiology and quantifies the financial affect on this organization. This retrospective cohort study was conducted through case and exposure identification, interviews, medical record reviews, and immunologic testing of blood specimens. Nine mumps cases resulted in 339 exposures, 325 (98%) among employees. During initial investigation, 186 (57%) of the exposed employees had evidence of mumps immunity. Physicians made up the largest group of noncompliers (55%) with mumps immunity testing. The cost to the institution was $262,788 or $29,199 per mumps case. The outbreak resulted in substantial staffing and financial challenges for the institution that may have been minimized with readily accessible electronic employee vaccination records and adherence to infection control recommendations.

## Abstract 2

Controlling the outbreak cost 4 times more than a routine prevention program.

Mumps, a highly contagious illness caused by a paramyxovirus, causes influenza-like symptoms and salivary gland swelling. Although rare, complications may include encephalitis, meningitis, orchitis, and oophoritis. The virus replicates within the upper respiratory tract and is transmitted through direct contact with respiratory droplets or saliva and through fomites. The incubation period ranges from 12 to 25 days; persons who contract mumps are considered infectious from 3 days before symptoms appear through 9 days after symptoms appear. Although no specific treatment exists, the disease is preventable through use of measles, mumps, rubella (MMR) vaccine usually provided to children ≈1 year of age with a booster dose administered before children start school. Clinical diagnosis is confirmed by laboratory testing that includes culture, serologic analysis, or real-time reverse transcription–PCR (RT-PCR) (*1**,**2*).

During January 1–October 7, 2006, 45 states reported 5,783 confirmed or probable cases to the Centers for Disease Control and Prevention (CDC). Six states, including Illinois, were responsible for 84% of reported cases. Mumps is generally more common among unvaccinated children, but nationally this outbreak occurred primarily among college-age persons (*3*). In Chicago, reported mumps cases began to increase in March 2006. By the end of the year, the Chicago Department of Public Health had 73 confirmed and probable cases. More of these cases were in an older age group (20–29 years) than was nationally observed (*4*).

Most healthcare worker (HCW) cases were concentrated in 1 hospital, Northwestern Memorial Hospital (NMH), Chicago Illinois, USA, which experienced ongoing transmission during April 23–May 23, 2006. The situation created resource and economic challenges to the organization. We examine the control and effects of this outbreak in a tertiary care center.

## Methods

### Clinical Setting and Patient Population

NMH is an 825-bed academic medical center. All adult patient care rooms are single occupancy; the neonatal intensive care unit (ICU) is multiple occupancy with 8 nurseries each housing 4–12 isolettes (self-contained incubator units, total of 67 isolettes). The patient cohort comprised all mumps case-patients and persons exposed to them at NMH during April 23–May 23, 2006. The NMH Institutional Review Board approved this study.

### Definitions

According to CDC, a clinical case of mumps is defined as acute onset of unilateral or bilateral tender, self-limited swelling of the parotid or other salivary glands lasting >2 days without other apparent cause. Confirmed cases are either laboratory confirmed or meet the clinical case definition and are epidemiologically linked to a confirmed or probable case. Probable cases meet the clinical case definition but are neither laboratory confirmed nor epidemiologically linked to another confirmed or probable case. Two probable cases that are epidemiologically linked are considered confirmed, even in the absence of laboratory confirmation (*3**,**5*). An exposure is defined as being within 3 feet of a person with mumps without use of appropriate personal protective equipment (*6*). A close contact is a visitor or family member exposed to a person with a confirmed or probable case. Persons are considered to have mumps immunity if they have documentation of receipt of 2 doses of mumps-containing vaccine, positive mumps immunoglobulin (Ig) G serologic results, or documentation of physician-diagnosed mumps (*4**,**7*). Persons with mumps serologies in the indeterminate range are considered nonimmune.

### Outbreak Investigation

The NMH Infection Control and Prevention Department (IC) investigated all mumps cases in an attempt to identify the index case and all persons who were exposed. Case-patients were placed in airborne infection isolation, as were exposed, nonimmune patients during their incubation period. Upon hospital discharge, case-patients and exposed patients were instructed to follow-up with the NMH Infectious Diseases Clinic (ID) or their primary care physicians. Similarly, patients discharged before recognition of exposure were contacted and referred to either ID or their primary care physician. IC sometimes needed to assign a provisional case status and to recommend a disposition before laboratory results were known. All cases were reported to the jurisdictional local health departments, and NMH provisional case status was retrospectively compared with the final case status assigned by the health departments.

According to NMH policy, all employees with communicable work-related illnesses or exposures are evaluated in the Corporate Health Department (CH). During this outbreak, employees with illnesses consistent with mumps were evaluated, furloughed through day 9 of their illness, and cleared by CH before returning to work. Ill employees were paid either through Workers’ Compensation (WC) after the first 3 days, for which employees are required by the Illinois State Workers’ Compensation Commission to use personal time off, or through the Short Term Injury and Illness Plan. Exposed employees were paid through a furlough account established by NMH during days 12–25 of the incubation period if nonimmune or while awaiting serologic test results. Employee compensation was managed through the NMH WC and Human Resources (HR) departments. Close contacts were referred to ID where immunity was determined at no charge to them.

Infection control data were collected through interviews and medical record review. Patient data were obtained from electronic medical records and employee data from written medical charts. Data included name, job title for employees; hospital location; exposure source for cases; and immunologic status, including previous receipt of MMR vaccine, history of mumps, and mumps serologic result with laboratory test date.

### Vaccine Program

Before 2003, only measles and rubella vaccination were routinely recorded in employee health records; thus, mumps vaccination status was often unavailable. To quickly assess mumps immunity during this outbreak, an intranet survey was created (SurveyMonkey, Portland, OR, USA) and made available to all employees. CH personnel reviewed survey results; results were not corroborated during the outbreak because of time constraints. To facilitate evaluation, counseling, and vaccination, nonimmune employees were seen either in CH, the Northwestern Medical Faculty Foundation Travel Medicine and Immunization Center, or in 1 of 2 satellite clinics established for this outbreak. Staff were classified as either high-risk caregivers (HRCs), low-risk caregivers (LRCs) or noncaregivers (NCs) to allow vaccine prioritization. HRCs were those who worked in areas where mumps cases were located or worked with pregnant or immunosuppressed patients. LRCs were persons who cared for patients in other inpatient or outpatient areas. To conserve resources, NCs were encouraged to seek evaluation with their primary care physicians but were not turned away if they sought evaluation at an NMH location.

### Laboratory Evaluation

NMH’s Immunology Laboratory performs mumps qualitative IgG antibody testing. Although most tests were performed in house, because of a low manufacturer’s supply of test kits, patient IgG testing was sent to a reference laboratory, and in-house testing was reserved for employees who were within 4 days of furlough. Turnaround time for the in-house test was decreased from 72 to 24 hours, and staffing was increased on weekends throughout the outbreak to ensure timeliness of test results. Reference laboratory turnaround time was 1–3 days. NMH’s Referred Testing Department sent serum to a reference laboratory for IgM and IgG antibody testing and buccal swabs to the Illinois Department of Public Health for RT-PCR.

### Financial Effects and Data Analysis

The financial effects were determined by tabulating the cost of personnel assistance and resource use. The cost of personnel assistance (i.e., lost productivity) was systematically provided by departmental leaders after the investigation. A total dollar value was assigned for each department by estimating the time spent by each employee on outbreak and exposure management. The cost for resources, represented by exact dollar amounts, includes medical evaluations, vaccines, laboratory evaluations, and employee compensation. Data for personnel assistance were stratified by department and aggregated to provide a total estimate. Data for resources were stratified by type of activity and aggregated to provide a total cost. Additionally, an estimate of the cost of maintaining a routine 2-dose MMR vaccination program and adequate employee medical records was calculated to compare with the cost of the outbreak. Data from 2008 were used for this calculation because 2008 was the first year NMH had complete financial records for the 2-dose MMR vaccination program. Financial data are rounded to the nearest dollar amount.

## Results

### Outbreak Investigation

**Figure 1:**
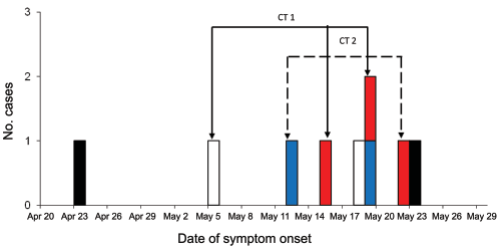
Epidemiology of 9 mumps cases at Northwestern Memorial Hospital, Chicago, Illinois, USA, April 23–May 23, 2006. Black bars, community-acquired cases among staff members; white bars, community-acquired cases among patients; red bars, secondary cases among staff members; blue bars, presumed work-related cases among staff members; CT1, first chain of transmission; CT2, second chain of transmission.

Nine mumps cases occurred at NMH, 7 among employees and 2 among inpatients (Table 1). Six were primary and 3 were secondary cases (Figure 1). Eight cases were symptomatic. Eight case-patients were women; the average age of all case-patients was 34 years (range 26–39 years). Two had documented receipt of 2 MMR vaccines, 2 had positive IgM serologic results, and none had documentation of prior mumps infection. Retrospectively, jurisdictional health departments assigned case status as follows: 4 confirmed, 3 probable, and 2 that could not be confirmed because even though both had clinical symptoms, 1 had negative laboratory results and the other had no known history of exposure.

**Table 1:** Epidemiology of 9 mumps cases, Northwestern Memorial Hospital, Chicago, Illinois, USA, 2006*

Case no.	Patient age, y/sex	No. MMR vaccine doses received	Clinical signs	Date of symptom onset	Serologic test results		DoH case disposition
					IgM	IgG	
1	35/F	1	Fatigue, unilateral facial swelling	Apr 24	–	+	Not a case

2	30/F	0	Fever, chills, stiff neck, bilateral facial swelling	May 5	–	+	Not a case
3	26/F	2	Fatigue, fever, headache, stiff neck, bilateral facial swelling	May 12	IND	+	Probable
4	39/F	0	Headache, sore throat, stiff neck, myalgias, bilateral facial swelling	May 15	+	IND	Confirmed
5	38/F	0	Flu-like illness, bilateral facial swelling	May 19	–	+	Probable
6	30/F	0	Headache, sore throat, myalgias, tender submandibular nodes	May 19	–	+	Probable
7	26/F	2	Sore throat, headache, bilateral facial swelling	May 22	–	+	Confirmed
8	44/F	1	Bilateral facial swelling	May 22	–	+	Confirmed
9	35/M	0	Asymptomatic	May 23	+	+	Confirmed

*MMR, measles, mumps, rubella; Ig, immunoglobulin; DoH, Department of Health; –, negative; +, positive; IND, indeterminate.

**Figure 2:**
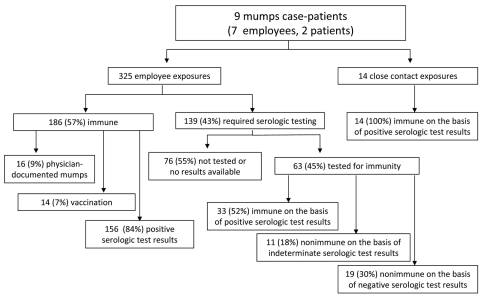
Immune status results among employees and close contacts exposed to 9 persons who had mumps, Northwestern Memorial Hospital, Chicago, Illinois, USA, 2006. For those deemed immune, immunity is grouped based on historical documentation of serologic status, mumps infection, or vaccination. All others were required to report for serologic testing during the outbreak; for those who complied with the required testing, immune status is provided.

**Figure 3:**
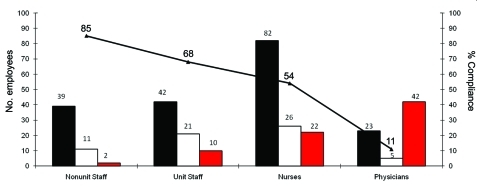
Mumps immunity status and compliance among employees, Northwestern Memorial Hospital, Chicago, Illinois, USA, 2006. Black bars, no. employees with history of immunity; white bars, no. employees who complied with required antibody titer testing; red bars, no. employees who did not comply with required antibody titer testing; black line, percent of employees in compliance. Unit staff consisted of nurse managers, secretaries, patient care technicians, clinical coordinators, and emergency department assistants; nonunit staff consisted of applications analysts, counselors, radiographers, resource coordinators, respiratory therapists, records specialists, safety technicians, patient escorts, housekeeping workers, and food services workers.

During the outbreak, 339 persons (325 employees and 14 close contacts) were reported as having been exposed to a person with mumps, resulting in an average of 38 exposures per case (Figure 2). Of the 325 employees, 186 (57%) were deemed immune: 16 (9%) with documented physician-diagnosed mumps, 14 (7%) with documented receipt of 2 doses of mumps-containing vaccine, and 156 (84%) with prior laboratory evidence of immunity. None of these employees required time off work because of the timely reporting of their mumps immune status. The remaining 139 (43%) employees required laboratory testing for immunity. Of these, 63 (45%) underwent testing, with serologic results as follows: 33 (52%) positive, 11 (18%) equivocal, and 19 (30%) negative. Overall, 219 (88%) of the 249 HCWs evaluated were immune to mumps. The remaining 76 (55%) employees who required testing for mumps immunity did not comply with CH evaluation (Figure 3). Of these persons, physicians made up 55%; registered nurses (RNs), 29%; unit staff, 13%; and nonunit staff, 3%. Fourteen close contacts required laboratory testing for mumps immunity, and all were immune.

A total of 59 employees were absent from work for 282 days for reasons that included having mumps, being nonimmune, and awaiting symptom evaluation or laboratory test results (Table 2). Employee time off work ranged from 1 to 24 days (average 5 days). RNs accounted for most of the work absences (n = 25, 42%) and took off the most days (94 days, 33%), followed by resident physicians (49 days, 17%). Furlough was the most used type of time off, with 229 days (81%), primarily for nonimmunity (178 days, 78%) followed by furlough awaiting serologic test results (51 days, 22%).

**Table 2:** Characteristics of employee work absences during mumps outbreak, Northwestern Memorial Hospital, Chicago, Illinois, USA, 2006*

Type of time off	Job title	No. employees	Days allowed
PTO	Registered nurse	1	3
	Patient escort	1	2
Short-term injury and illness plan	Unit secretary	1	6
WC	Patient care technician	1	24
	Registered nurse	1	10
WC and PTO	Registered nurse	2	8
Furlough			
Because of nonimmunity	Registered nurse	9	48
	Physician	5	49
	Unit staff†	9	42
	Nonunit staff‡	7	39
Because of pending titer results	Registered nurse	12	25
	Unit staff	8	20
	Nonunit staff	2	6
Total		59	282

*PTO, personal time off; WC, workers’ compensation †Nurse managers, secretaries, patient care technicians, clinical coordinators, emergency department assistants. ‡Applications analyst, counselor, radiographer, resource coordinator, respiratory therapist, records specialist, safety technician, patient escort, environmental services, food services.

During April 1–June 31, 2006, 416 mumps IgG serologies were performed at NMH; 58 IgM and 207 IgG serologic results were sent to a reference laboratory. Twenty-nine buccal swabs were sent to the Illinois State Laboratory for mumps RT-PCR, and only 2 were positive, both from outpatients unrelated to the institutional outbreak.

### Vaccine Program

**Figure 4:**
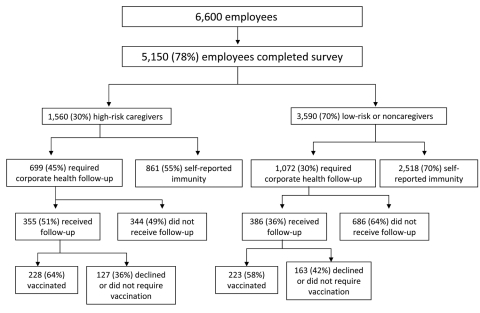
Survey results of self-reported mumps immunity among workforce, Northwestern Memorial Hospital, Chicago, Illinois, USA, 2006. Results are categorized by high-risk caregivers, those who worked in areas where mumps cases were located or worked with pregnant or immunosuppressed patient populations; low-risk caregivers, those who cared for patients in other inpatient or outpatient areas; or noncaregivers. Compliance with corporate health evaluation and vaccination for those who did not report immunity are also shown.

Of the 6,600 NMH employees, 5,150 (78%) completed the intranet survey to assess their mumps immunity (Figure 4). Of these, 1,560 (30%) were HRCs and 3,590 (70%) were LRCs or NCs. Ninety-one percent of HRCs and 74% of LRCs and NCs completed the survey. Of the HRCs who completed the survey, 699 (45%) required additional follow-up; however, only 355 (51%) complied. Of those who complied, 228 (64%) received vaccination. In comparison, 1,072 (30%) LRCs and NCs required additional follow-up, and 386 (36%) complied. Of these, 223 (58%) received vaccination. Overall, 127 (36%) of HRCs and 163 (42%) of LRCs or NCs either declined or did not require vaccination. The average time for employee evaluation in CH was 30–45 minutes, and the 2 satellite clinics operated for 177 hours. From April 20 through September 1, 2006, CH administered a total of 755 MMR vaccinations, 451 to survey participants.

### Financial Effects

The estimated cost of personnel assistance during the mumps outbreak was $66,432, led by IC at $36,746 (55%) (Table 3). The largest contribution from a hospital unit was the neonatal ICU at $6,624 (10%). The actual cost of resources was $196,356. The largest resource contributors were HR resulting from compensation for employee time off work at $91,318 (47%) and CH at $56,256 (27%) from time required for medical record review. The total cost of the outbreak was $262,788, representing a 3:1 ratio of resource to personnel costs. Cost per mumps case was $29,199.

**Table 3:** Financial effects of mumps outbreak, Northwestern Memorial Hospital, Chicago, Illinois, USA, 2006*

Type of expense and department	Cost, US$*
Personnel	
Human resources	1,066
Infection control and prevention	36,746
Laboratories	7,312
Medical administration	1,500
Nursing units	10,808
Patient escort	1,200
Risk management	300
Environmental and occupational safety	7,500
Total personnel cost	66,432
Resources	
Corporate health	56,256
Human resources employee compensation	91,318
Infectious diseases clinic	1,000
Laboratories	6,842
Travel medicine and immunization center	2,240
Vaccination program	38,700
Total resource cost	196,356
Total cost to hospital	262,788

*Rounded to the nearest dollar.

In comparison, in 2008 maintaining a routine 2-dose MMR vaccination program and adequate employee medical records cost ≈$66,025. This figure represents the annual number of new employees (n = 978), all of whom required a $30 medical record review and the annual number of MMR vaccinations given (n = 667) at $55 each. Thus, the cost of controlling the mumps outbreak was 4× the cost of maintaining a routine MMR prevention program.

## Discussion

Transmission of mumps can occur within hospitals, but outbreaks with secondary transmission such as the one at NMH are rarely reported (*8**,**9*). One of the most widely reported incidents of nosocomial transmission occurred during a community mumps outbreak in Tennessee in 1986–1987 (*8*). Although only a small number of cases were nosocomially transmitted, this in-hospital outbreak illustrates the threat that mumps and other illnesses can pose to patients and HCWs (*8**,**10*).

Although investigators have quantified the impact of nosocomial mumps outbreaks, in-depth analysis of resource use during a large-scale nosocomial mumps outbreak has not been published (*9,**11*). Analysis of this outbreak assigned a cost for the resources used and the personnel affected. Most of the resource cost was attributable to HR for compensation for staff work absences and to CH for employee health record review. Additionally, personnel most affected were from IC and from the neonatal ICU, the inpatient unit requiring the most staffing substitutions. In comparison, a nosocomial mumps outbreak in Utah in 1996 reported the total cost of the outbreak in an inpatient pediatric facility was $3,140, substantially lower than our cost (*9*). Examination of these 2 outbreaks, however, indicates that they are not comparable. The Utah facility was much smaller than NMH (45 vs 825 beds), had fewer staff, and had only 2 cases. The smaller work environment and magnitude of the outbreak posed less opportunity for exposure to an infected person and required far fewer resources for outbreak control. In contrast, a neonatal ICU outbreak of infection with respiratory syncytial virus, an illness spread through a similar route, involving 9 infants was reported to have cost >$1.15 million (*12*). Although the number of cases is similar to ours, the increased cost of the outbreak of infection with respiratory syncyntial virus reflects the need for intensive care and expensive postexposure prophylaxis (*12*). These discrepancies highlight the need for organizations to conduct and report detailed disease-specific analyses to assist similar institutions planning for resource use during outbreak prevention and control.

At NMH, the lack of complete and easily retrievable employee health records contributed substantially to the overall outbreak cost. Until recently, only documentation of rubella and measles immunity was required and mumps immune status was often not recorded; additionally, vaccination information was not available electronically. During the outbreak, the need to rapidly evaluate the mumps immunity of our workforce would have required review of >6,000 employee health records, a task not deemed practical to prevent ongoing disease transmission and excessive employee furlough. This challenge led to development of an electronic survey to query employees about their mumps immunity. Although obviously suboptimal, this approach allowed CH to focus on record review for employees who either did not know their status or did not respond to the survey and to manage the ongoing vaccine campaign. This situation is not unique to NMH. Analysis of previous mumps outbreaks identified complete and easily retrievable employee vaccination records as an integral step in reducing the resource and financial costs to the hospital (*8**,**9**,**13**,**14*). If employee health information was complete and accessible, more than one fourth of our outbreak cost might have been averted.

Vaccination of HCWs is vital to mumps outbreak prevention. Although numerous outbreaks have occurred in populations with only 1-dose vaccine coverage, the national mumps outbreak of 2006 occurred during the highest 2-dose vaccine coverage in the United States at 87%, making this the first large-scale national mumps outbreak associated with 2-dose vaccine failure. The estimated herd immunity threshold for mumps ranges from 88% to 92%, and during the outbreak at NMH, 88% of our evaluated workforce reported mumps immunity. The experience nationally and at our institution supports the concept that an increased level of group-specific immunity may be required to prevent transmission in settings in which close or prolonged contact occurs, particularly in crowded conditions, such as those within healthcare institutions (*9**,**13**,**15*). The possibility of vaccine failure highlights the need to maximize immunity among HCWs with 2 doses of MMR vaccine and to address the age of administration of an MMR booster or the addition of a third vaccine dose to prevent future outbreaks (*13**–**15*).

Our outbreak highlights the inaccuracies that can exist in mumps case recognition, resulting in both underestimation and overestimation of disease. Cases can be underestimated because patients are contagious for days before symptoms appear, and up to one third of patients never develop symptoms but can still spread disease. Notably, 1 exposed, asymptomatic employee underwent IgG and IgM testing and was positive for IgM. Fortunately, no secondary cases are known to have resulted from exposure to this person. In addition, overestimation can occur when presumptive case status is assigned on the basis of clinical presentation before laboratory results are available. At NMH, 2 probable cases could not be confirmed by the health departments. These cases led to additional exposure evaluations. Although prompt initiation of infection control measures is vital to control a mumps outbreak, investigators should be aware of the challenges in accurate case recognition.

The lack of laboratory resources also increased the cost of the outbreak. The on-site laboratory testing facility required increased staffing to complete timely serologic testing and later had a shortage of testing kits. The need to send specimens to a reference laboratory delayed test results and led to assignment of presumptive case status on the basis of symptoms resulting in potentially unnecessary exposure evaluations. In addition, the hospital had to furlough exposed employees whose immune status was unknown until serologic results were available.

The lack of compliance with IC recommendations for exposure evaluation and vaccination was evident primarily among physicians. This reaction was similar to that during a mumps outbreak in 1987 at the Chicago Mercantile Exchange in which the intense nature and competitiveness of the profession encouraged employees to work while ill (*16*). The reasons for lack of compliance at NMH, particularly among physicians, are unknown, but the urgent nature of the profession is expected to have played a major role. That some employees minimized the risk for exposure or thought the follow-up process was too cumbersome also has been speculated. Another finding was the discovery of a few persons who claimed exposure to benefit from time off work. Cooperation between CH, IC, WC, and HR led to detection and management of these rare instances.

When examining the types of employee compensation provided, an inherent inequality was established. Ill employees were not fully compensated for their work absence (67% of the employee’s average weekly wage after taking 3 days of personal time off). These employees were required to take WC, and the rate of compensation is set according to the Illinois Workers’ Compensation Act (www.state.il.us/Agency/IIC/act.pdf). In contrast, exposed employees were compensated by a system specifically established for this outbreak by the hospital because WC will not cover such costs. These persons were compensated 100% of their salary. This unbalanced system of reimbursement may require reevaluation for future outbreaks so that ill persons do not feel penalized or fail to self-disclose illness.

This study has several limitations. First, recall bias may have occurred, particularly when departmental leaders retrospectively estimated personnel costs. Second, the reliability of self-reported information obtained through interviews and the intranet survey regarding mumps immunity was not validated during the outbreak and may have contributed to either overestimation or underestimation of mumps immunity in our workforce. Finally, the findings of this study may not be generalizable because all healthcare institutions are unique environments.

We examined the effects of the 2006 national mumps outbreak within a healthcare institution. Our cost of >$262,000 makes a strong business case for healthcare organizations to improve infectious diseases prevention and control strategies. A comprehensive program that consists of maintaining complete electronic employee health records, identifying cases and employee exposures rapidly, enforcing compliance with infection control recommendations, and developing plans to alleviate laboratory shortages is of paramount importance for outbreak control. Reports of detailed epidemiologic and financial analyses of infectious disease outbreaks can facilitate emergency preparedness and response planning for comparable healthcare organizations.
